# Porous Carbon Materials Obtained by the Hydrothermal Carbonization of Orange Juice

**DOI:** 10.3390/nano10040655

**Published:** 2020-04-01

**Authors:** Francesco Veltri, Francesca Alessandro, Andrea Scarcello, Amerigo Beneduci, Melvin Arias Polanco, Denia Cid Perez, Cristian Vacacela Gomez, Adalgisa Tavolaro, Girolamo Giordano, Lorenzo S. Caputi

**Affiliations:** 1 Surface Nanoscience Group, Department of Physics, University of Calabria, I-87036 Rende, Cosenza, Italy; francesco.veltri@unical.it (F.V.); francesca.alessandro@fis.unical.it (F.A.); andrea.scarcello@fis.unical.it (A.S.); 2 UNICARIBE Research Center, University of Calabria, I-87036 Rende, Cosenza, Italy; melvin.arias@intec.edu.do (M.A.P.); dcid@pucmm.edu.do (D.C.P.); cvacacela@yachaytech.edu.ec (C.V.G.); 3 INFN, Sezione LNF, Gruppo Collegato di Cosenza, Via P. Bucci, I-87036 Rende, Cosenza, Italy; 4 Department of Chemistry and Chemical Technologies, University of Calabria, I-87036 Rende, Cosenza, Italy; amerigo.beneduci@unical.it; 5 Laboratorio de Nanotecnología, Área de Ciencias Básicas y Ambientales, Instituto Tecnológico de Santo Domingo, Av. Los Próceres, Santo Domingo 10602, República Dominicana; 6 Escuela de Ciencias Naturales y Exactas, Pontificia Universidad Católica Madre y Maestra, Autopista Duarte Km 1 1/2, Santiago de los Caballeros 51000, República Dominicana; 7 CompNano, School of Physical Sciences and Nanotechnology, Yachay Tech University, Urcuquí EC-100119, Ecuador; 8 Research Institute on Membrane Technology (ITM-CNR), University of Calabria, I-87036 Rende, Cosenza, Italy; a.tavolaro@itm.cnr.it; 9 Department of Environmental and Chemical Engineering, University of Calabria, I-87036 Rende, Cosenza, Italy; ggiordaunical@yahoo.it

**Keywords:** hydrothermal carbonization (HTC), carbon microspheres, KOH activation

## Abstract

Porous carbon materials are currently subjected to strong research efforts mainly due to their excellent performances in energy storage devices. A sustainable process to obtain them is hydrothermal carbonization (HTC), in which the decomposition of biomass precursors generates solid products called hydrochars, together with liquid and gaseous products. Hydrochars have a high C content and are rich with oxygen-containing functional groups, which is important for subsequent activation. Orange pomace and orange peels are considered wastes and then have been investigated as possible feedstocks for hydrochars production. On the contrary, orange juice was treated by HTC only to obtain carbon quantum dots. In the present study, pure orange juice was hydrothermally carbonized and the resulting hydrochar was filtered and washed, and graphitized/activated by KOH in nitrogen atmosphere at 800 °C. The resulting material was studied by transmission and scanning electron microscopy, Raman spectroscopy, X-ray photoelectron spectroscopy, X-ray diffraction, and nitrogen sorption isotherms. We found porous microspheres with some degree of graphitization and high nitrogen content, a specific surface of 1725 m^2^/g, and a pore size distribution that make them good candidates for supercapacitor electrodes.

## 1. Introduction

Thermal conversion is nowadays a strongly investigated route to convert natural precursors in carbon nanomaterials to be used in supercapacitor electrodes [1,2,3,4,5,6]. The decomposition of biomass is often obtained by pyrolysis in the absence of oxygen, giving rise to the preferential production of biochar (solid products), bio-oil, or gases, depending on final temperature and thermal environment [7,8,9,10,11]. In recent years, wet pyrolysis, also called hydrothermal carbonization (HTC), has gained increasing interest due to the relative simplicity of the process, including the fact that biomass does not need to be dried before treatment, avoiding the energy consumption due to the drying process [12,13,14,15]. In fact, water contained in natural precursors is part of the medium in which hydrothermal reactions take place. The solid product of HTC is named hydrochar to distinguish it from biochar obtained by pyrolysis. With respect to dry pyrolysis, HTC has different advantages: lower working temperatures, lower ash production, a higher density of functional groups containing oxygen in the solid product.

In HTC, biomass is subjected to autogenous pressure in a confined environment in the presence of subcritical water, i.e., water in the liquid phase at temperatures between 100 and 374 °C [16,17,18]. Subcritical water acts as reactant and catalyst: its dielectric constant decreases from 78 F/m at 25 °C and 0.1 MPa to 14 F/m at 350 °C and 20 MPa [19]. The ionic product increases from 10^−14^ at ambient conditions up to about 10^−12^ in the subcritical range, increasing the solubility of hydrophobic organic compounds [20,21,22]. Different investigations have shown that in the HTC of biomass, the degradation of carbohydrates, lignin, lipids, and proteins gives rise to hydrochars with a very low specific surface area (SSA) [23,24,25,26,27]. Therefore, physical or chemical activation is necessary to make these hydrochars suitable to be used as electrodes of supercapacitors. 

Orange peel and orange pomace were investigated as feedstocks for the production of hydrochars. Erdogan et al. [28] studied the hydrochars and the aqueous phase produced by the HTC of orange pomace at various temperatures and residence times. Fernandez et al. [29] activated hydrochars obtained by the HTC of orange peels to assess them as adsorbent of contaminants. Physical and chemical activation resulted in specific surface areas in the 300–620 m^2^/g range. Orange peel waste was treated by HTC and then mixed with lignin, cellulose, and hemicellulose to modify the quantity and properties of resulting hydrochar [30]. It was shown that cellulose and hemicellulose increased the density of microspheres on the surface of hydrochars, and a maximum SSA of 27.35 m^2^/g was found for lignin-added hydrochar. To the best of our knowledge, the HTC of orange juice was performed only to synthesize carbon quantum dots. Sahu et al. [31] performed HTC at 120 °C of orange juice mixed with ethanol and separated carbon dots from coarse particles by centrifugation. In a very similar experiment, Kumar et al. [32] obtained carbon quantum dots by HTC at 120 °C of orange juice mixed with ethylenediamine. 

In this work, we present the HTC of pure orange juice at mild temperatures, without adding any chemicals in the hydrothermal environment. Hydrochar production was optimized and chemical activation by KOH was performed to obtain a porous carbon material. We obtained microspheres with high SSA and optimal pore distribution that should ensure rapid ion diffusion in suparcapacitor application.

## 2. Materials and Methods 

Fresh oranges (*Citrus Sinensis*) were purchased from a local market in Rende, Italy. The oranges were squeezed and the juice was filtered by a fine mesh stainless steel sieve to separate the liquid from the skins, seeds, pulp, and stems. Analytical-grade KOH and HCl were purchased from Sigma-Aldrich and used as received.

A total of 30 ml of pure orange juice was transferred into a PTFE-lined 60-ml autoclave and treated at 180 °C for 6 h. After HTC, the autoclave was allowed to cool down at room temperature, and the solid product was washed with ultrapure water, filtrated and dried overnight at 80 °C, obtaining a fine powder. KOH was dissolved in deionized water and the powder was dispersed in the KOH solution, with a KOH/powder mass ratio of 3. The mixture was then dried and heated in a tube furnace at 800 °C in 800 ml/min nitrogen flux for 30 min. To remove K compounds, after being cooled at room temperature, the final product was thoroughly washed with diluted HCl (10 vol%), followed by deionized water until pH 7.0 was reached. Finally, the sample was recovered and dried in air at 80 °C. The yield of the final carbon powder was 1.4% with respect to the initial orange juice mass. To measure the ash content, the sample was dehydrated in an oven at 110 ± 1 °C until a constant weight was achieved. The dried sample was incinerated at 550 ± 5 °C in a muffle furnace until complete combustion, and the ash content was determined by weight on the dry sample. The morphology and size of the powders were studied by transmission electron microscopy (TEM) using a JEM 1400 Plus microscope (JEOL Ltd., Tokyo, Japan) with 80 kV accelerating voltage, using Formvar-coated copper grids, and by scanning electron microscopy (SEM), by a EVO 10 microscope (CARL ZEISS AG, Oberkochen, Germany) with 5 kV accelerating voltage. The hydrochars were metallized with gold due to their low conductivity. X-ray photoelectron spectroscopy (XPS) was performed in an ultra-high vacuum system equipped with a Phoibos 100 hemispherical analyzer (VSW Ltd., Manchester, UK), with non-monochromated Al-kα radiation. Quantitative elemental composition was done by the flash combustion of the sample in a CHNS Analyzer Flash 2000 (Thermo Scientific, Waltham, MA, USA). The combustion products (CO_2_, N_2_, H_2_O and SO_2_) were separated by an analytical chromatographic column and quantitated by a thermal conductivity detector against an external calibration standard (Thermo Scientific Aspartic Acid Standard) with the linear calibration method. Raman spectra were measured by a NRS-500 spectrometer (Jasco Corp., Tokyo, Japan) with a 532-nm laser wavelength, 0.3 mW, 100× objective, with samples deposited on glass substrates. Fourier-transform infrared (FT-IR) spectroscopy was applied by a FTIR Spectrum One system (Perkin Elmer Inc., Waltham, MA, USA) within the wavenumber range 650–4000 cm^−1^, with a resolution of 0.5 cm^−1^. Powder X-ray diffraction (XRD) analyses were performed by a D8 Discover diffractometer (Bruker Corp., Billerica, MA, USA) with Cu Kα radiation between 10° and 60°, with a scan rate of 0.02° min^−1^. Nitrogen adsorption/desorption isotherms were obtained by a ASAP2020 system (Micromeritics Instrument Corp., Norcross, GA, USA) at liquid nitrogen temperature (−196 °C). The SSA and the pore size distribution were determined by the Brunauer–Emmett–Teller (BET) and Barrett–Joiner–Halenda (BJH) method, respectively.

## 3. Results

**Figure 1:**
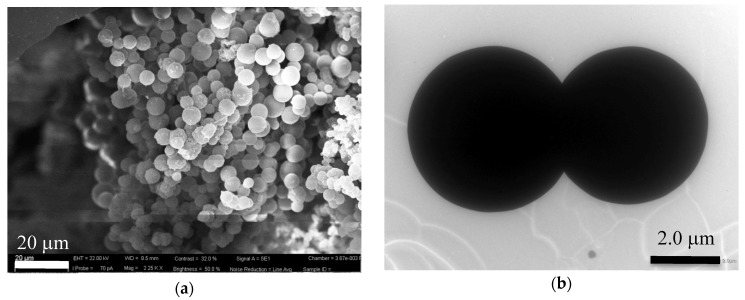
Typical (**a**) scanning electron microscopy (SEM) and (**b**) transmission electron microscopy (TEM) images of the hydrochar obtained after 6 h of the HTC of orange juice at 800 °C.

**Figure 2:**
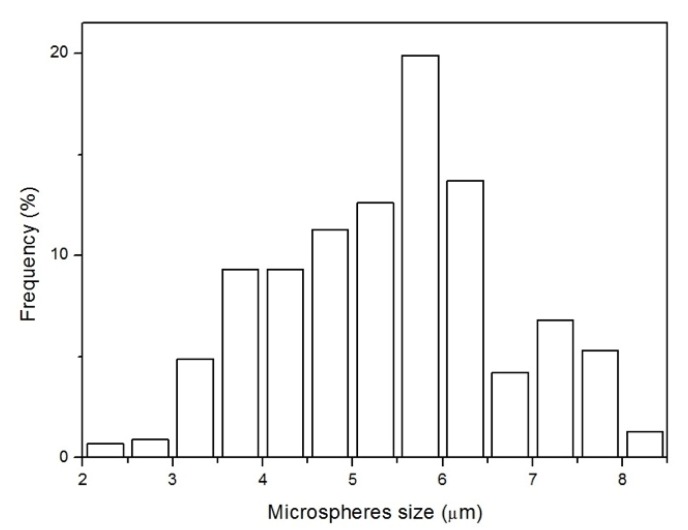
Size histogram of the hydrochar microspheres, giving an average value and standard deviation of 5.4 and 1.3 μm, respectively.

The hydrochar obtained from orange juice was in form of microspheres, as shown in the SEM and TEM images in Figure 1. Statistical analysis of the size of the spheres gave the histogram shown in Figure 2, from which we calculated an average value and a standard deviation of 5.4 and 1.3 μm, respectively.

**Figure 3:**
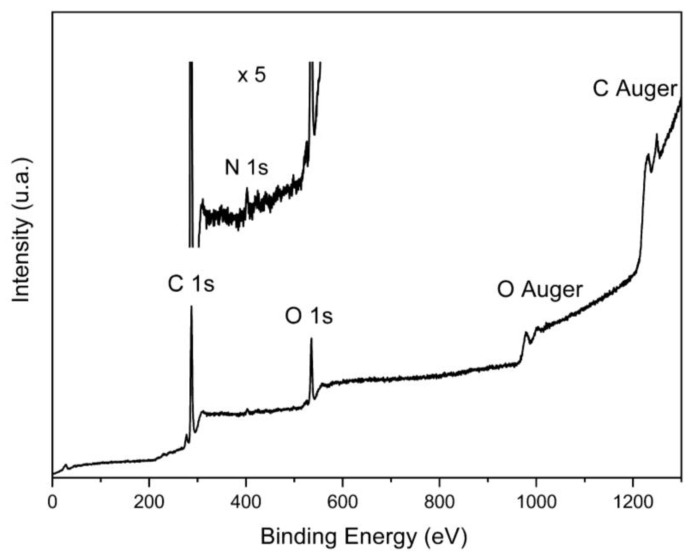
XPS survey of the hydrochar obtained from orange juice, where the presence of carbon and oxygen with traces of nitrogen is evident. The inset shows a magnification of the region containing the N 1s peak on the same energy scale of the complete spectrum.

Figure 3 shows the XPS spectrum of the microspheres. It is evident that the carbonization process gives rise to a material made mainly of carbon and oxygen, with some nitrogen, evidenced in the inset of Figure 3. The spectrum shows no traces of other impurities. 

**Figure 4:**
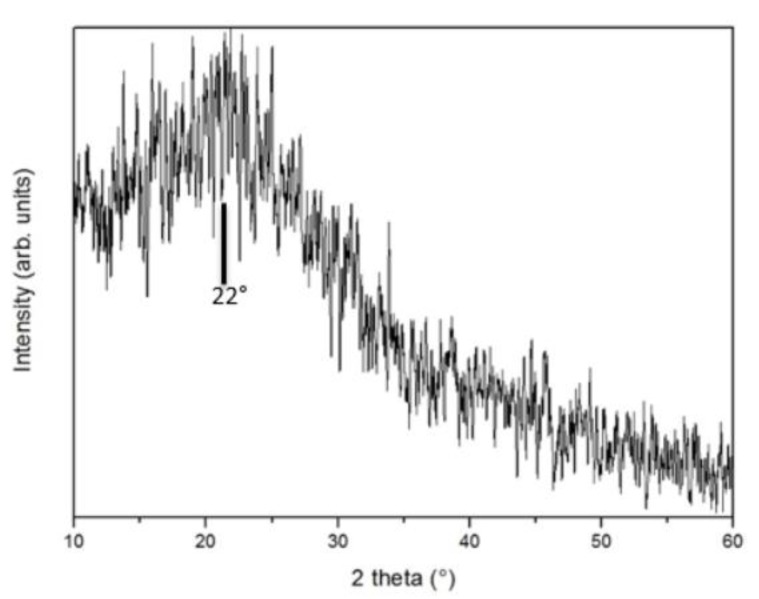
XRD profile of microspheres, showing a wide peak that can be related to the (200) peak of graphite.

The XRD profile of the microspheres is shown in Figure 4. The presence of the broad peak centered at about 22° is consistent with the (002) diffraction planes in carbonaceous materials with graphitic character.

**Figure 5:**
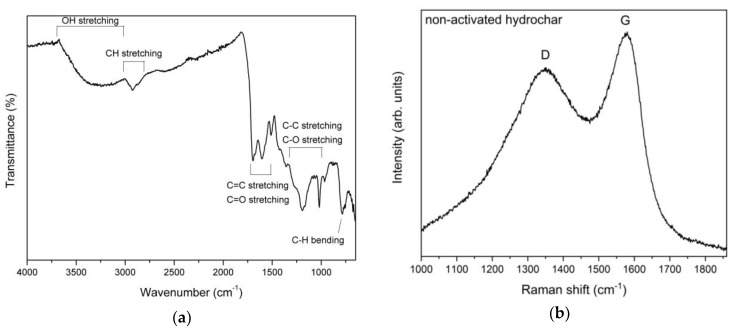
Spectroscopic characterization of hydrochar microspheres: (**a**) FT-IR spectrum; (**b**) Raman spectrum, with *I_D_*/*I_G_* = 0.83.

The surface functional groups of hydrochar microspheres were examined by FT-IR. The typical spectrum is shown in Figure 5a, where bands are assigned on the basis of the literature [33,34]. The Raman spectrum, reported in Figure 5b, has the typical shape of char materials due to the presence of polycyclic aromatic hydrocarbons [35,36,37]. The peaks G and D are characteristic of graphitic materials and are due to the C–C stretching and aromatic breathing mode, respectively [38]. The D mode is defect-induced and is used as an indicator of the degree of crystalline order in graphene-like materials. The *I_D_*/*I_G_* peak intensity ratio has a value of 0.83. The porosity of hydrochar microspheres was studied by nitrogen adsorption/desorption experiments. Before activation, the BET SSA was 12 m^2^ g^−1^.

**Figure 6:**
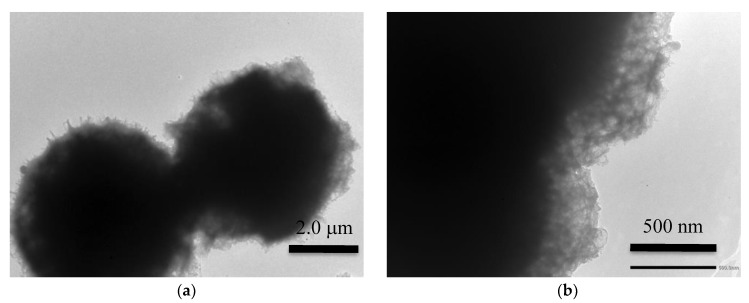
(**a**) TEM image showing that the spherical shape was retained after graphitization/activation. In the image taken at higher magnification (**b**), the induced porosity is evident in the peripheral area of the spheres.

The morphology of microspheres was strongly affected by KOH graphitization/activation. In Figure 6, we report TEM images after activation, which show that the spherical shape was retained, with an induced porosity clearly visible in the peripheral areas. The ash content of the activated sample was below 0.08 weight %. CHNS elemental analysis showed no traces of sulphur in the hydrochar and in the activated sample. C, H, and N weight % are given in Table 1. 

**Figure 7:**
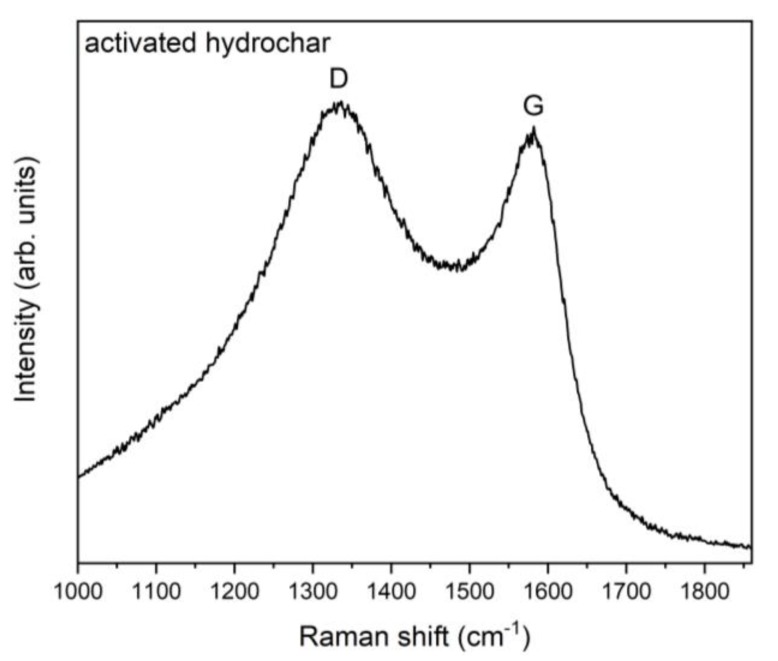
Raman spectrum of activated microspheres, with *I_D_*/*I_G_* = 1.17.

The Raman spectrum of activated microspheres is reported in Figure 7. Comparison with the spectrum in Figure 5b shows that the *I_D_*/*I_G_* intensity ratio has increased, the new value being 1.17.

**Figure 8:**
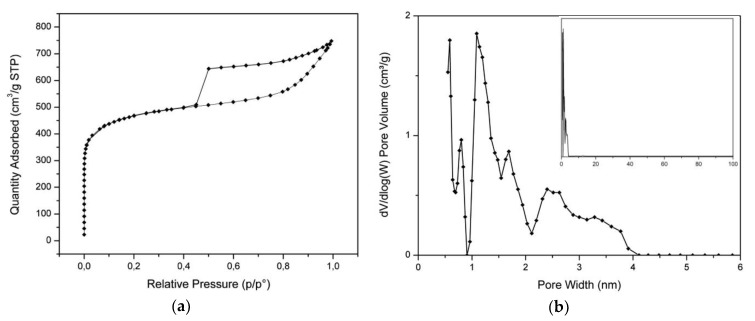
(**a**) Nitrogen sorption isotherms of the hydrochar microspheres, and (**b**) pore size distribution. The inset in (**b**) shows the distribution up to 100 nm.

Figure 8 shows the nitrogen adsorption/desorption results for the activated sample. The isotherm in Figure 8a exhibits the type-IV shape (IUPAC classification), with hystheresis loop due to capillary condensation in mesopores [39]. The calculated BET SSA was 1724 m^2^/g. The pore size distribution, shown in Figure 8b, indicates the absence of macropores up to 100 nm.

## 4. Discussion

The nucleation of clusters resulting from the hydrolysis of the carbon-containing constituents of orange juice gives rise to spherical particles with an average radius of about 5 μm. The SEM and TEM images in Figure 1 show spheres with a well-defined surface. The XPS spectrum in Figure 3 shows the absence of elements other than carbon, oxygen, and nitrogen. CHNS elemental analysis of the hydrochar reveals a weight % of 76.21, 5.38, and 5.65 for carbon, hydrogen, and nitrogen, respectively. The X-ray diffraction in Figure 4 indicates that the microspheres contain partially graphitized particles. The width of the (200) peak, centered at about 22°, is very likely due to a wide range of interplanar distances, higher with respect to graphite, in which the peak is centered at 26.7°. The partially graphitic character of microspheres is confirmed by the Raman spectrum in Figure 5b. The presence of the defect-activated D peak, although showing a large width compared to the spectrum of defected graphite [40], is indicative of the presence of aromatic rings. In graphitic materials, the *I_D_*/*I_G_* peak intensity ratio is used to estimate the degree of crystallinity. In the present case, *I_D_* and *I_G_* have to be intended as the peak heights. This is the more reasonable choice because in hydrochars obtained by the carbonization of biomass, the broadening of the D peak is due to contributions coming from distorted clusters with ring orders other than 6. It has been shown that, in these materials, the D width can be associated to ring-related breathing and Kekulé modes in benzoperylene, benzopyrene, coronene, perylene, pyrene, and naphthalene clusters [41]. Therefore, the information about less distorted rings is contained in the D height rather than its area [42]. 

The surface of the microspheres is covered by oxygen functionalities, as demonstrated by the FT-IR spectrum in Figure 5b. Peaks can be interpreted as being due to the presence of hydroxyl, carboxyl, carbonyl, quinone, and ester species [43,44,45,46].

Hydrochar microspheres exhibit very low porosity: the SSA measured by nitrogen adsorption/desorption is of the order of tens of m^2^/g.

KOH activation does not change the overall shape of the hydrochar, which appears still in form of spheroidal particles with an irregular surface that can be interpreted as being due to the induced porosity (Figure 6). The elemental analysis shows that, after activation, the weight % of carbon, hydrogen, and nitrogen is 76.36, 0.75, and 13.67 respectively. Therefore, graphitization/activation does not cause a significant change in the carbon content. On the contrary, the final hydrogen content is very low, and the nitrogen percentage increases significantly. Considering the measured ash content (<0.08 weight %), we calculate an oxygen weight % of 9.14 in the activated sample. The high nitrogen content could be important in view of a possible use of the material in supercapacitor electrodes. In fact, it has been shown that, in porous carbon materials derived from biomass, N-doping improves the oxidation stability and the electrochemical performance in the perspective of their use in supercapacitor electrodes [47,48]. Liu et al. [49] studied N-doped porous carbon derived from banana peel and showed that nitrogen induces a noticeable improvement in both the specific capacitance and the cycle life.

The Raman spectra of the non-activated and activated sample show an increase in the *I_D_*/*I_G_* peak intensity ratio, from 0.83 to 1.17. In graphenic-graphitic materials, an increase in the ratio is usually interpreted as being due to an increased density of defects. However, in the case of chars, which are made of small clusters containing aromatic rings, the interpretation is opposed. It has been shown by Ferrari and Robertson [42] that for crystallites with a lateral size lower than 20 Å, an increase in the *I_D_*/*I_G_* intensity ratio is related to an improved level of order. In fact, for small crystallites, a high density of defects can destroy aromatic rings, lowering the intensity of the breathing D mode, while the G peak does not suffer a decrease of the same extent, being related to the C–C stretching mode. In the case of chars obtained by the carbonization of biomass, the clusters containing distorted carbon rings with different ring orders, much like those considered in ref [41], have sizes below 20 Å. Therefore, the *I_D_*/*I_G_* increase very likely indicates an increased structural order generated by activation, rather than an increased disorder. As a matter of fact, treatment with KOH at 800 °C in nitrogen atmosphere cannot be simply considered as an activation, but rather a graphitization-activation. Ma et al. [50] treated the hydrochar microspheres obtained by the hydrothermal carbonization of tree leaves in a very similar way. XRD measurements before and after graphitization-activation showed some evidence of enhanced graphitization after the KOH treatment. Our findings and those of Ma et al. are reasonable because hydrochars have a low degree of graphitization, and treatment at 800 °C in the presence of KOH causes the destruction of part of the material, but very likely the remaining carbon material has a graphitization degree higher with respect to starting microspheres.

The BET SSA calculated from nitrogen adsorption/desorption experiments is 1725 m^2^/g, which is not in the higher range of the SSAs observed in activated hydrochars but is noticeably higher with respect to the hydrochars obtained by the HTC of orange peel [29]. However, the SSA value is not the only aspect to be considered to assess porous carbon materials as potential electrodes in supercapacitors. A crucial role is played by the pore size distribution, which must exhibit both micropores to maximize the amount of charge and then the energy density and mesopores to facilitate the flux of electrolite ions, increasing the power density. Raymundo-Pinero et al. investigated the performances of supercapacitor electrodes made of porous carbons obtained by the pyrolysis of seaweeds [51]. By tuning the porosity, they obtained the best specific capacity for nanocarbons with a moderate specific surface.

The activated carbon microspheres obtained in this work have a high density of micropores, as demonstrated by the pore size distribution in Figure 8b, and a mesopore structure clearly observed in the same curve, which gives rise to the typical hystheresis in the isotherm in Figure 8a. It is interesting to note that the pore size distribution we measured is very similar to that reported by Wei et al. [52], where a huge specific capacitance was obtained in double-layer symmetric capacitors.

## 5. Conclusions

Orange juice is not considered to be a waste, and this is probably the reason why it has not been considered as a potential feedstock for hydrochar production. However, low-quality oranges do not have market in southern Italy and their cost can be roughly estimated to be a few tens of euros per ton. Given the high quality of the final product in terms of specific surface area, pore size distribution, and nitrogen content, it can be concluded that the production of porous carbon by the hydrothermal carbonization of orange juice followed by graphitization/activation by KOH appears to be a greatly interesting route to develop efficient and low-cost supercapacitor electrodes. 

## Figures and Tables

**Table 1:** Results of the CHNS elemental analysis of the hydrochar and of the final activated sample.

Sample	C ^1^	H	N
Hydrochar	76.21	5.38	5.65
Activated sample	76.36	0.75	13.67

^1^ All quantities are given in weight %.
